# Evaluating the Relative Perceptual Salience of Linguistic and Emotional Prosody in Quiet and Noisy Contexts

**DOI:** 10.3390/bs13100800

**Published:** 2023-09-26

**Authors:** Minyue Zhang, Hui Zhang, Enze Tang, Hongwei Ding, Yang Zhang

**Affiliations:** 1 Speech-Language-Hearing Center, School of Foreign Languages, Shanghai Jiao Tong University, Shanghai 200240, China; zhang.my@sjtu.edu.cn (M.Z.); zhanghui_helen@126.com (H.Z.); tangenze@sjtu.edu.cn (E.T.); 2 Department of Speech-Language-Hearing Sciences and Masonic Institute for the Developing Brain, University of Minnesota, Minneapolis, MN 55455, USA

**Keywords:** babble noise, lexical tone, emotional prosody, masking

## Abstract

How people recognize linguistic and emotional prosody in different listening conditions is essential for understanding the complex interplay between social context, cognition, and communication. The perception of both lexical tones and emotional prosody depends on prosodic features including pitch, intensity, duration, and voice quality. However, it is unclear which aspect of prosody is perceptually more salient and resistant to noise. This study aimed to investigate the relative perceptual salience of emotional prosody and lexical tone recognition in quiet and in the presence of multi-talker babble noise. Forty young adults randomly sampled from a pool of native Mandarin Chinese with normal hearing listened to monosyllables either with or without background babble noise and completed two identification tasks, one for emotion recognition and the other for lexical tone recognition. Accuracy and speed were recorded and analyzed using generalized linear mixed-effects models. Compared with emotional prosody, lexical tones were more perceptually salient in multi-talker babble noise. Native Mandarin Chinese participants identified lexical tones more accurately and quickly than vocal emotions at the same signal-to-noise ratio. Acoustic and cognitive dissimilarities between linguistic prosody and emotional prosody may have led to the phenomenon, which calls for further explorations into the underlying psychobiological and neurophysiological mechanisms.

## 1. Introduction

In human communication, prosodic features of the spoken language fulfill important linguistic and socio-affective functions. Emotional prosody refers to the prosodic expression of the emotional state of the speaker [1], whereas linguistic prosody relates to the use of prosody to specify linguistic information [2]. While linguistic and emotional prosodies serve different communicative functions, both are acoustically characterized by variations in fundamental frequency (also referred to as F0 or pitch), intensity, duration, and voice quality [3,4,5]. Recognizing linguistic tone and emotional prosody is crucial for effective communication, as these cues provide information about the speaker’s intent, mood, and emotional content of their message.

In tonal languages such as Mandarin Chinese, pitch variations play a crucial role in distinguishing word meanings at the syllabic level, forming phonemic contrasts known as lexical tones [6]. Despite their importance for conveying phonological and semantic contrasts, lexical tones share some characteristics with prosody, such as their suprasegmental pitch variations and larynx-based articulation [7], and are therefore considered an important constituent of linguistic prosody [8]. Mandarin Chinese comprises four lexical tones differentiated by their pitch contours: high and flat (Tone 1), rising (Tone 2), falling and then rising (Tone 3), and falling (Tone 4). The perception of Mandarin lexical tones largely relies on fundamental frequency (F0) [9,10], with F0 contour and F0 height being the primary acoustic cues used to distinguish between the four tones [11,12,13,14]. Although the co-varying intensity and duration parameters in Mandarin speech provide supplementary/redundant perceptual cues [9,15], there is evidence that manipulating duration and amplitude may have little effect on lexical tone perception (e.g., [16]).

Listening conditions play a significant role in how people perceive and interpret linguistic as well as emotional prosody. Everyday communication often takes place in noisy environments, such as bustling streets, crowded cafes, busy offices, or even during social events. These conditions can range from quiet environments with minimal background noise to noisy settings with various auditory distractions. In noisy contexts, individuals may encounter difficulties in accurately perceiving and distinguishing linguistic tone and emotional prosody due to reduced auditory clarity. This can lead to misinterpretations, misunderstandings, increased effort and cognitive load, and challenges in effective communication. The robustness of Mandarin lexical tone perception in adverse listening conditions has been well documented [17,18,19,20,21,22]. In the comparable signal-to-noise ratio (SNR) conditions for both steady-state and fluctuating maskers, Mandarin lexical tone recognition performances were found to be better than English sentence recognition [23]. Wang and Xu [22] further verified this phenomenon by observing that speech-shaped noise and multi-talker babble with various numbers of talkers had less impact on Mandarin lexical tone perception than on recognition of English vowel-consonant-vowel syllables, words, or sentences. The high robustness of lexical tones relative to other linguistic segmental elements (especially those in non-tonal languages) has been attributed to listeners’ additional use of frequency-modulation information (referred to as temporal fine structure by Qi et al. [21]) in tone perception. This feature is reported to be particularly resistant to background noise degradation [18,24,25,26].

Unlike lexical tones whose perception is highly related to the listener’s linguistic knowledge and experience [27,28,29], emotional prosody conveys a broad range of emotional states, among which basic emotions (typically including happiness, sadness, anger, fear, disgust, and surprise [30]) can be recognized across cultures [31,32]. Basic emotional prosody displays a more universal feeling [33], and vocal emotion communication is constrained largely by biological factors [34] and governed by universal principles across languages and cultures [35,36]. However, these findings and views were primarily based on non-tonal languages. Later cross-linguistic comparisons have shown that despite the universality of emotional expressions, the specific mechanisms of utilizing acoustic cues for encoding emotions in various languages are still different (e.g., [37]). Similar to lexical tones, acoustic parameters such as pitch, duration, and intensity have been found to be important for emotion identification [33,38,39,40,41]. Many studies additionally pointed out the significance of voice quality features in distinguishing emotions (e.g., anger and happiness [42,43]). In tonal languages, the existence of a lexical tone system may restrict the use of pitch for paralinguistic purposes [44], thus highlighting the importance of other acoustic cues, particularly voice quality, for conveying vocal emotions [37].

Most investigations into how background noise affects emotion recognition have focused on improving automatic emotion recognition using speech enhancement and artificial intelligence algorithms (e.g., [45,46]). However, recent studies have started to explore how background noise influences emotion perception in human listeners (e.g., [24,47,48,49,50,51]). For instance, Parada-Cabaleiro et al. [48] investigated the effects of three types of background noise (white, pink, and Brownian) on emotional speech perception and found that all types of noise negatively impacted performance, with pink noise having the most significant effect and Brownian the least. Scharenborg et al. [47] examined the influence of babble noise on verbal emotion perception in both native and foreign languages, while Zhang and Ding [49] explored how background babble noise affected emotion identification in unisensory and multisensory settings. The findings of these studies consistently demonstrate that background noise, particularly babble noise, can have detrimental effects on emotion perception.

Two theoretical accounts exist with opposing claims on the relative salience or functional weight of linguistic versus emotional prosody. According to the “functional load” hypothesis [52], lexical tones in tone languages carry a high functional load with phonemic status equivalent to that of vowels. Ross et al. [53] extended this idea to examine emotional prosody in Mandarin Chinese, in comparison with English, and found that the use of tone in a language limits the extent to which F0 can be freely used to signal emotions. These findings suggest that linguistic prosody may be more salient than emotional prosody in tonal languages where tone is used to distinguish between different words. However, Xu [54] demonstrated that various aspects of prosody are encoded by different mechanisms that rely on F0 for different purposes, implying that tonal languages may not have a limited capacity for intonation for linguistic or paralinguistic functions. In contrast, the social signaling theory [34,55] posits that emotional prosody is crucial for nonverbal communication and conveys information about the speaker’s emotional state, personality, social identity, intentions, and attitudes towards the listener. While both emotional prosody and linguistic prosody are important for social signaling, emotional prosody may be more salient because it communicates critical social and affective information.

While there is theoretical debate on the relative salience of linguistic and emotional prosody, few studies have empirically investigated their relative perceptual resilience under adverse listening conditions. Recent studies have shown that white noise has a greater impact on word recognition than emotional prosody recognition in English [24]. However, whether these results generalize to tonal languages such as Mandarin Chinese remains unclear. Moreover, previous studies have used different testing paradigms for assessing word/sentence recognition versus emotional prosody recognition (i.e., open-set tests for word/sentence recognition vs. forced-choice close-set tests for emotional prosody recognition), rendering the identification of emotions much simpler [21,22]. In addition, although white noise has been used in previous research, using multi-talker babble noise, which is commonly encountered in everyday listening environments [56,57], may provide a more ecologically valid measure of the impact of background noise on prosody perception. Researchers have observed that Mandarin lexical tone recognition remains robust even in adverse listening conditions, with performance plateauing at *N* = 8 in all SNR conditions when using multi-talker babble noise [22].

Given that everyday communication frequently occurs in noisy environments, understanding how people cope with these challenges and how they adapt their communication strategies is essential. The present study aimed to investigate the relative perceptual resilience of Mandarin lexical tones and emotional prosody in background multi-talker babble noise. We hypothesized that lexical tones would be more perceptually salient than emotional prosody under adverse listening conditions with masking babble noise. Understanding the relative salience of linguistic and emotional prosody in different listening conditions is crucial for ensuring effective communication and providing insights into improving communication strategies, enhancing educational experiences, and gaining a deeper understanding of human cognitive and emotional processes. Furthermore, within the sphere of Mandarin Chinese studies, this endeavor holds particular significance. Mandarin Chinese is a tonal language, where subtle changes in pitch patterns, known as lexical tones, carry distinct meanings. This linguistic feature adds a layer of complexity to prosody perception in adverse listening conditions, setting Mandarin apart from non-tonal languages. Given that Mandarin is one of the most widely spoken languages globally [58], understanding how its unique prosodic elements are perceived in noisy environments is essential not only for Mandarin speakers but also for cross-linguistic prosody research. By delving into the specific challenges faced by Mandarin speakers, this study contributes not only to advancing our understanding of prosody perception but also to deepening our comprehension of the intricate cognitive and emotional processes involved in Mandarin Chinese speech perception.

## 2. Methods

### 2.1. Participants

We recruited participants through online campus advertisements to participate in our study. To meet the selection criteria, participants had to be native Mandarin Chinese speakers who predominantly used Mandarin in their daily lives, with no history of speech, language, or hearing impairments, and no reported psychological or neurological conditions. From this pool of eligible individuals, we randomly selected 40 participants (21 females and 19 males) with a mean age of 22.19 years (±2.76 SD) to take part in our research. Audiological screening, which included pure-tone assessments ranging from 0.25 to 8 kHz (≤20 dB HL) [59], confirmed that all participants had normal hearing. Prior to the experiment, each participant provided written informed consent, and they received compensation for their participation following the study.

### 2.2. Stimuli

Eight monosyllabic interjections, 嘿, 啊, 哎, 呀, 哈, 诶, 咳, and 哦 (International Phonetic Alphabet [xeɪ], [a], [aɪ], [ja], [xa], [eɪ], [xaɪ], and [ɔ]), were chosen to carry emotional prosody and lexical tones. We chose monosyllabic interjections out of two major considerations. One is that the carriers of emotional prosody and lexical tones should be the same to enable legitimate comparisons between them, and the other is about ecological validity. Interjections are important devices in conversations to express mental or emotional states [60], and monosyllables in Mandarin Chinese can be pronounced with one of the four lexical tones [61]. It is therefore ecologically valid to use monosyllabic interjections as the carriers in this experiment. Each monosyllable was produced with four emotions (happy, sad, angry, and calm) and four lexical tones (level tone, Tone 1; rising tone, Tone 2; dipping tone, Tone 3; and falling tone, Tone 4) within a soundproof chamber by two amateur actors (one female and one male), both native speakers of Mandarin Chinese. This resulted in the creation of 128 sound clips, generated from eight interjections, spanning eight categories (four emotions and four lexical tones), and featuring the contributions of two distinct actors. High-quality recordings were acquired using a Neumann U87 Ai condenser studio microphone (Georg Neumann, Berlin, Germany) in conjunction with a Fireface UFX soundboard (RME Fireface; RME Inc., Meridian, ID, USA). The recordings were digitized at a 44,100 Hz sampling rate, maintaining a 16-bit amplitude resolution. Subsequently, they underwent a normalization process, ensuring a consistent peak value (90%), which was achieved through Adobe Audition CC (Adobe Systems, San Jose, CA, USA). Thirty native Mandarin Chinese who did not take part in the current study were invited to validate the stimuli with the identification accuracy for each category being at least 90%.

The pitch, intensity, and duration measures of the prosodic stimuli are shown in Table 1. Pitch and intensity measurements were conducted on the vowel portion of the stimuli. The onset and offset of a pitch or intensity contour were determined by the beginning and cessation of periodicity of the waveform. For Tone 4 productions, since a substantial number of irregular cycles, indicating creakiness, was observed at the offset, the endpoint in such productions was determined by the last identifiable cycle. The contour was divided into 100 intervals of equal duration. F0 values in Hz and intensity values in dB were then obtained at the 101 time points and missing points in the middle caused by creakiness were interpolated using ProsodyPro [62] in Praat 6.0.37 [63]. The F0 and intensity values were manually checked for accuracy.

**Figure 1:**
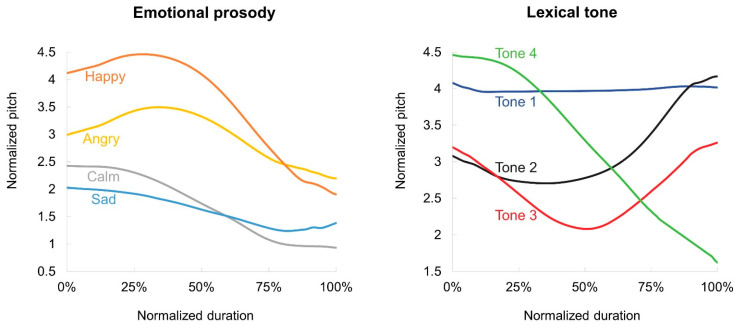
The pitch contours of the emotional prosody and the lexical tone stimuli. All prosodic contours were normalized to have the same duration, and the F0 values were log-transformed. (Tone 1: high and flat; Tone 2: rising; Tone 3: falling and then rising; Tone 4: falling).

The productions of lexical tone and emotional prosody stimuli were normalized using the *T*-value logarithmic transform to account for interspeaker variability in F0 range,

(1) $$T=\left[\left(lgX-lgL\right)/\left(lgH-lgL\right)\right]\times 5,$$

where *X* represents the observed F0, and *H* and *L* are the maximum F0 and minimum F0, respectively, of the speaker [64]. Figure 1 displays the normalized pitch contours of the emotional prosody and lexical tone stimuli, averaged across all speakers and tokens. The pitch contours of the lexical tone stimuli adhere to the canonical contour of the four lexical tones in Mandarin Chinese [65] and the pitch contours of the emotional prosody stimuli closely resemble those reported by Li [66].

The stimuli were presented in two listening conditions (i.e., quiet and noise). For the noise condition, we used an eight-talker babble created by Chen et al. [67] as the background noise. It was created by mixing eight emotionally neutral sentences produced by eight native Mandarin Chinese speakers. The babble noise underwent normalization to achieve a consistent peak value of 90% using Adobe Audition CC (Adobe Systems, San Jose, CA, USA). It was then introduced into the target stimuli with a signal-to-noise ratio (SNR) set at −13 dB. This SNR level was carefully determined during pilot testing to strike a balance between avoiding ceiling performance and effectively masking the target sounds. The babble noise onset occurred approximately 500 ms before the commencement of the target sound and persisted for approximately 500 ms after the target ceased.

### 2.3. Procedure

The experiment was conducted in a sound-attenuating room with the participant seated at approximately 60 cm from an LCD monitor. We used Experiment Builder (Version 2.3.38; SR Research) for stimulus presentation. The sounds were presented binaurally using high-fidelity circumaural headphones (Sennheiser HD 280 Pro; Sennheiser, Old Lyme, CT, USA) at a comfortable level (70 dB SPL). There were two tasks, emotion recognition and tone recognition. In each task, the participants listened to a total of 128 stimuli (8 [interjections] × 4 [categories] × 2 [actors] × 2 [conditions]) that were presented in two blocks. The tasks and blocks were counterbalanced across all participants to ensure equitable distribution. Each block comprised 64 trials, arranged in a pseudorandom order. Participants were instructed to provide their responses with both speed and precision, achieved by pressing one of four designated response keys, each corresponding to either one of the four emotional categories or the four lexical tones. While the mappings between emotions/tones and keys were counterbalanced across participants, they remained consistent for each individual throughout the entire experiment. Prior to commencing the experiment, we ensured that participants comprehended the overall procedures and fully grasped the key-category correspondences. Each block started with a practice phase, encompassing four trials. To proceed to the test phase, participants were required to achieve 100% accuracy in the practice phase, ensuring a clear understanding of the task. Adequate breaks were interspersed between blocks to mitigate potential fatigue.

### 2.4. Statistical Analyses

To compare the masking effects of babble noise on emotional prosody and lexical tones, we applied a series of generalized linear mixed-effects models in R (Version 4.1.3) with the *lme4* package [68]. Accuracy and reaction time were entered as dependent variables, respectively. For the analysis of accuracy, binomial response data were used and a binomial distribution with a logit link function was employed. For the analysis of reaction time, a gamma distribution with a log-link function was implemented [69]. Before analyzing reaction time data, we preprocessed them by excluding incorrect responses and responses over 2 SDs from the mean [70,71]. Within-subject variables, task (emotion and tone) and listening condition (quiet and noise) were entered as categorical fixed factors. Speakers and items were included as a random intercept term to account for the subject- and item-level variability. Tukey’s post hoc tests in the *emmeans* package [72] were implemented for pairwise comparison when there was a significant main effect or interaction effect. *p*-values were obtained by likelihood ratio tests of the full model with the effect in question against the model without the effect in question. The full models with intercepts, coefficients, and error terms are, respectively, represented in Formulas (1) and (2) in Supplemental Material S1.

## 3. Results

Supplemental Tables S1 and S2 summarize the detailed results of the generalized linear mixed-effects models for identification accuracy and reaction time.

### 3.1. Accuracy

**Figure 2:**
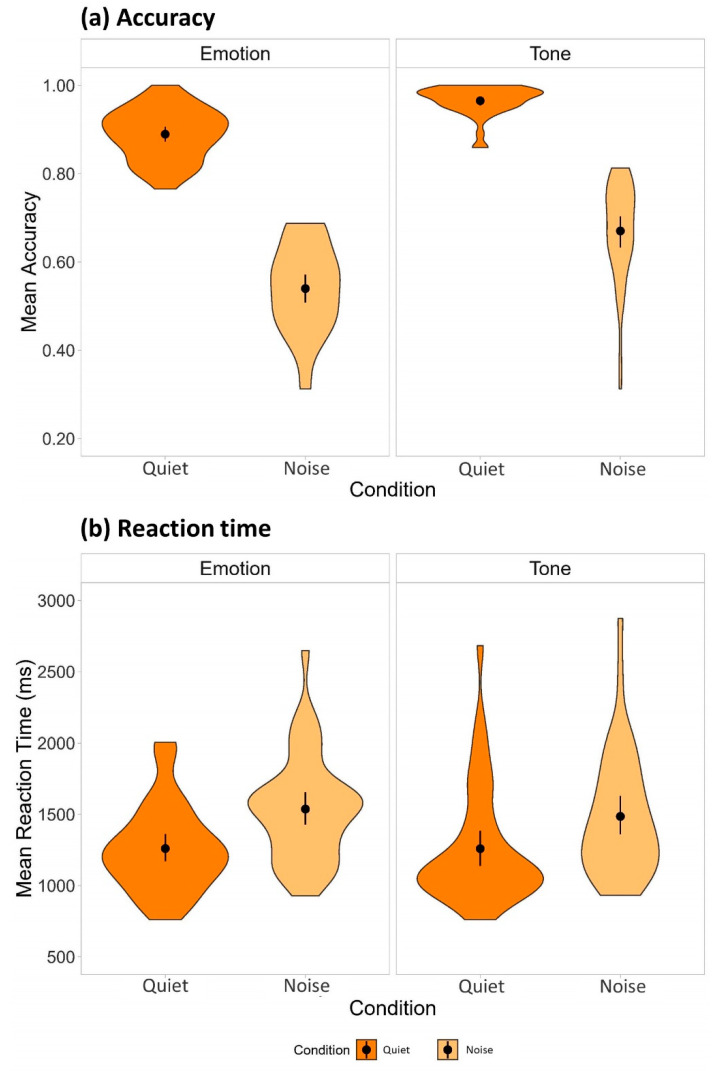
Mean (**a**) accuracy and (**b**) reaction time in the emotion and lexical tone recognition tasks. Mean accuracy and reaction time are displayed in the violin plots with data distribution shapes indicated by the density plots, mean values represented by the black dots, and 95% confidence intervals shown by the error bars.

Figure 2a illustrates the mean proportion correct in the quiet and noise listening conditions for the two tasks. Generalized linear mixed-effects analyses revealed significant main effects of task, χ^2^(2) = 199.46, *p* < 0.001, Cohen’s *w* = 2.23, and condition, χ^2^(2) = 1752.9, *p* < 0.001, *w* = 6.62, and a significant interaction between task and condition, χ^2^(1) = 27.75, *p* < 0.001, *w* = 0.83. In the emotion recognition task, listeners achieved 35.9% ± 2.1% lower accuracy in the noise condition compared with the quiet condition (${\hat{\beta }}_{3}$ = 2.03, *SE* = 0.08, *z* = 26.45, *p* < 0.001, *d* = 2.39). In the lexical tone recognition task, adding the same background babble noise led to a 29.9% ± 2.3% reduction in the identification accuracy (${\hat{\beta }}_{3}$ = 2.74, *SE* = 0.12, *z* = 23.42, *p* < 0.001, *d* = 3.23). Lexical tone stimuli elicited 7.0% ± 0.9% more accurate responses than emotional prosody stimuli in the quiet condition (${\hat{\beta }}_{3}$ = −1.26, *SE* = 0.13, *z* = −9.97, *p* < 0.001, *d* = −1.48), with the tone versus emotion gap further increased to 12.9% ± 1.4% in the noise condition (${\hat{\beta }}_{3}$ = −0.54, *SE* = 0.06, *z* = −9.16, *p* < 0.001, *d* = −0.64).

### 3.2. Reaction Time

For the reaction time data, we excluded incorrect responses (mismatch between listener responses and the intended emotion/lexical tone category conveyed by the speaker in that particular trial; 7.23% for the quiet condition and 39.90% for the noise condition) and responses over 2 SDs from the mean (5.16% for the quiet condition and 3.09% for the noise condition). Figure 2b illustrates the mean reaction time in the two listening conditions for the two tasks. Generalized linear mixed-effects analyses showed significant main effects of task, χ^2^(2) = 20.16, *p* < 0.001, *w* = 0.71, and condition, χ^2^(2) = 408.73, *p* < 0.001, *w* = 3.20, and a significant interaction between task and condition, χ^2^(1) = 5.17, *p* = 0.02, *w* = 0.36. In the emotion recognition task, response time was increased by 279.8 ± 21.8 ms in the noise condition compared with the quiet condition (${\hat{\beta }}_{3}$ = −0.20, *SE* = 0.013, *z* = −15.44, *p* < 0.001, *d* = −0.27). Within the lexical tone recognition task, there was also a significant increase by 215.3 ± 18.6 ms in the noise condition relative to the quiet condition (${\hat{\beta }}_{3}$ = −0.16, *SE* = 0.012, *z* = −13.35, *p* < 0.001, *d* = −0.22). Participants responded at 86.8 ± 21.0 ms faster to the lexical tone stimuli than to the emotional prosody stimuli in the noise condition (${\hat{\beta }}_{3}$ = 0.06, *SE* = 0.014, *z* = 4.20, *p* < 0.001, *d* = 0.08), despite no significant difference between the two tasks in the quiet condition (*p* = 0.374).

## 4. Discussion

The current study investigated the relative perceptual salience of Mandarin lexical tones and emotional prosody in background multi-talker babble noise. In line with our prediction, the accuracy and reaction time data showed a perceptual advantage of Mandarin lexical tones over emotional prosody. Specifically, native Mandarin Chinese speakers achieved higher identification accuracy and responded faster to the lexical tone stimuli, with these differences further amplified in the presence of masking babble noise. These findings align well with previous studies that have highlighted the robustness of Mandarin lexical tones to background noise (e.g., [22]). Our results support the “functional load” account, which emphasizes the prominence of lexical tones over emotional prosody in tonal languages such as Mandarin Chinese. We propose that the observed perceptual advantage of lexical tones can be attributed to both acoustic and cognitive differences between lexical tones and emotional prosody, as well as the specific characteristics of the masking babble noise used in this study.

Multi-talker babble noise produces two kinds of masking effects, that is, energetic masking (EM) and informational masking (IM). EM derives from the reduced audibility of the target because of the overlap in time and frequency between the signal and the masker, which is believed to influence processing from the level of the cochlea. IM arises from the similarity between the target and the masker despite the clear audibility of both and involves competition for resources in the central auditory system [73,74]. The mechanisms behind EM and IM can be explained through a framework based on auditory object formation and auditory object selection [75]. Object formation involves segregating the target source from maskers and object selection concerns selectively listening to the target while ignoring competing maskers. In our study, the eight-talker babble noise brought considerable difficulty in object formation with its high noise level but little in object selection due to its unintelligibility [76]. Hence, it brought about significant obstacles to extracting the acoustic features of the target stimuli but little lexical interference or competition for neural resources [77].

The acoustic characteristics of emotional prosody in Mandarin Chinese may have rendered its object formation more difficult in the presence of background noise. While the perception of Mandarin lexical tones depends majorly on pitch, the acoustic correlates of Mandarin emotional speech involve less contribution from pitch but more a crucial role of voice quality [78]. Since fundamental frequency is found to be more resistant to noise degradation than phonation-related cues [79,80], the extraction of acoustic cues for emotional prosody presumably would become harder than that for lexical tones in adverse listening conditions. Moreover, the acoustic realization of vocal emotions in Mandarin is characterized by its multidimensionality [37]. Due to the restricted paralinguistic use of pitch to accommodate the lexical tone system, other acoustic dimensions, including duration, intensity, and voice quality, are strengthened in compensation [37,81]. This may well increase the listeners’ difficulty in integrating the necessary acoustic cues for emotion identification in the context of high-level background noise. Thus, the disadvantages in both extracting and integrating acoustic cues for emotional prosody together contributed to its less successful object formation in background noise. Admittedly, sources of difficulty could come from object selection—the other challenge of cocktail party listening. In our study, eight-talker babble noise introduced little linguistic interference because of its unintelligibility and thus might not have created a big obstacle for lexical tone perception. Rather, the speech elements in the masker could be competing for auditory attention, which would affect lexical tone recognition.

Another consideration is the psycho-cognitive differences between the two types of prosody. For each trial, listeners need to make cognitive evaluations of the target prosody [82] in attaching a label to the perceived prosodic expression. The cognitive evaluation process for emotional prosody might be less automatic than that for lexical tones because of the additional conceptual processing in the categorization of emotional expressions [83]. Numerous studies have documented a quite early acquisition and establishment of lexical tone categories [84,85] but not so for emotion perception. Emotional expressions are perceived in terms of valence in early development and become associated with discrete emotion categories over time as children learn emotion words [86]. It has been shown that the emotional specialization for vocal prosody occurs even later in adolescence [87]. Challenging listening environments may hinder the conceptual labelling for emotional prosody recognition and thus become especially disadvantageous to emotion perception.

Additionally, lexical tone recognition involves a strong top-down process [88,89,90] where prior language experience and linguistic knowledge promote the recognition of a pitch contour as a certain tone category [91]. As shown in Figure 1, the pitch contours of the lexical tone stimuli in this study exhibit a high degree of conformity to the canonical pitch contours of Mandarin Chinese lexical tones. The smaller reduction in the identification performances for lexical tones (as a type of linguistic prosody) thus aligns with the consensus view that top-down linguistic knowledge works well in compensating for the reduced informativeness of the bottom-up signals [92,93].

Both lower-level sensory and higher-level cognitive distinctions may be at work to influence the disparity of noise influences on the two types of prosody. That is, it might be more difficult to extract and integrate the acoustic cues of emotional prosody in babble noise due to its strong employment of noise-susceptible phonation-related parameters and its acoustic multidimensionality. It is also possible that the cognitive evaluation of emotional prosody before judgment involved additional conceptual processing that might be impeded in adverse conditions, whereas lexical tone recognition in noise may benefit from top-down facilitation driven by language experience, which can compensate for the signal loss from noise masking.

Our results are also consistent with the neurolinguistic view that prosody is processed in a hierarchical manner, that is, from sensory processing via auditory integration toward evaluative judgments [4,82,94]. This hierarchical 3-stage model of prosody perception may also be applicable in adverse listening environments. It remains unclear how emotional prosody and lexical tones resemble and differ from each other in terms of their neural underpinnings and mechanisms. In this regard, it is important to examine neural activations to determine at which stages of speech prosody perception involve more acoustic processing and at which stages the processing of functional classes (affective vs. linguistic) of speech prosody emerge. Do the two aspects happen discretely, or do they interact throughout the perception of prosodic information? Do emotional prosody and lexical tone perception in degraded conditions reflect the same functional hemispheric specialization as that in ideal listening environments? Answers to these questions may emerge when we disentangle the psychobiological and neurophysiological overlapping and non-overlapping between lexical tone processing and emotional prosody processing in both quiet and noise conditions.

In comparing our findings with existing research in the field, our results align with several relevant studies (e.g., [18,22]) while also displaying some disparities when contrasted with previous similar investigation. It has been found that segmental information in speech materials is more susceptible to noise degradation when compared to suprasegmental information [24]. On the surface, this observation may seem incongruent with our findings. However, a deeper examination reveals several key factors contributing to this discrepancy. Firstly, previous similar research primarily focused on English, a non-tonal language, where segmental information pertained to individual words. In contrast, our study is centered around Mandarin Chinese, a tonal language, where segmental information significantly revolves around lexical tones. Mandarin lexical tone perception relies heavily on fundamental frequency (F0) [9,10], which distinguishes it from other linguistic segmental elements, such as consonants. Consequently, it is reasonable that the impact of background noise on the perception of Mandarin lexical tones differs significantly from that on English segmental elements. Secondly, the divergence in noise types employed between prior research, often utilizing white noise, and our study, featuring multi-talker babble noise, introduces another layer of complexity. A growing body of research highlights the differential effects of various noise types on the perception of speech materials [95,96]. Therefore, the varying noise environments in these studies could account for the disparities observed in the susceptibility of segmental information to background noise. In light of these contextual nuances, our results provide valuable insights into the distinctive challenges posed by different languages and noise conditions in the realm of speech prosody perception. This underscores the importance of considering language-specific and noise-specific factors when interpreting the resilience of segmental and suprasegmental information in adverse listening conditions.

There are limitations in this study. First, based on pilot testing, we chose only one specific SNR level for the noise condition to answer our hypothesis. It remains to be explored how variations in noise-induced degradation would affect the relative robustness of emotional prosody and lexical tones in background babble noise. Second, we chose only one type of noise (eight-talker babble) and did not incorporate other types of noise. Differences in the maskers may differentially impact lexical tone recognition and emotional prosody recognition. Third, communication involves more than just spoken words. Rather, it is a complex interplay of various sensory and modal cues that work together to convey meaning, emotions, and intentions. Our experimental protocol does not take into consideration the multimodal and multisensory nature of communication, which is essential for effective interpersonal interactions [5,97,98]. Speech communication is a holistic experience that involves integrating auditory, visual, tactile, and contextual cues to comprehend both the literal content and the emotional nuances of the message. This concept is particularly relevant in cross-cultural communication, where different cultures may rely on different modal cues to convey meaning and emotions, especially in adverse listening conditions. Moreover, this understanding has implications in fields such as psychology, linguistics, and human–computer interaction, where researchers seek to create more realistic and natural communication models and technologies.

Our study provides an initial step for the comparison between the perception of emotional prosody and lexical tones in adverse listening conditions. Several lines can be pursued in the future. The first is to determine the role of language experience and linguistic knowledge in perceiving prosodic information in noise. Native tonal-language speakers may perform better in identifying linguistic prosody due to their tonal category knowledge. Different cultures may place varying degrees of emphasis on linguistic tone and emotional prosody [99,100,101]. Studying how these cues are interpreted across cultures and contexts can enhance intercultural communication and reduce misunderstandings. It would be enlightening to examine and compare the perception of emotional prosody and lexical tones in noise by non-tonal language speakers or Chinese-as-a-second-language learners in comparison with native speakers of Chinese. The second is to assess the relative masking effects of IM and EM on the two types of prosody by manipulating their proportion in background babble noise, which may be subject to influences of aging and aging-related hearing loss and cognitive decline [102,103,104]. The contribution of IM can be adjusted by varying the number of talkers in the babble noise or using speech samples from a non-tonal language unknown to the listeners to create babble noise. Speech-shaped noise can also be added for comparison purposes. The impact of noise on emotional prosody and lexical tones can depend on the type of noise and specific acoustic features of the speech signal. Babble or speech-shaped noise, for example, may have a greater effect on emotional prosody because they can disrupt the rhythm and timing of speech. Similarly, certain speech features such as pitch range or duration may be more critical for emotional prosody than for lexical tones, and therefore more susceptible to interference from noise. Furthermore, different SNR levels could be used to vary the degree of EM, which is typically greater at lower SNR levels [105]. Thirdly, it is important to consider how emotional prosody and lexical tones may interfere with each other [53,106,107]. Emotional prosody can make it harder to discern the subtle pitch differences that distinguish different lexical tones, while exaggerated or artificially manipulated lexical tones can alter the perception of emotional prosody. The extent of interference can depend on the specific task and context and may be symmetric or asymmetric. Individual differences in language proficiency, cognitive processing strategies, and attentional control can also affect the degree of interference. Additionally, the role of vowels/syllables may also need to be taken into consideration in this interaction. Finally, utilizing neurophysiological and neuroimaging techniques such as ERP and fMRI to record neural activity during the processing of emotional prosody and lexical tones in noise would help capture acoustic, psychobiological, and neurofunctional similarities and differences between various categories of prosodic information [7,108,109,110,111,112]. This approach can provide valuable insights into how the brain processes and distinguishes between different types of prosody, which have implications for individuals with perception/production difficulties with speech prosody [113,114,115,116,117].

## 5. Conclusions

Given that everyday communication frequently occurs in noisy environments, understanding how people cope with these challenges and how they adapt their communication strategies is essential. This study investigated the perception of Mandarin lexical tones and emotional prosody in quiet and in background multi-talker babble noise. Compared with emotional prosody, Mandarin lexical tones were more perceptually salient in noise. The higher salience of lexical tones in babble noise is in line with the distinctions between the two types of prosody at the three stages of the hierarchical model for prosody perception, which provides the impetus for further exploring the neural substrates of emotional prosody perception and lexical tone perception as well as their temporal and regional overlapping. Further investigations spanning a broader spectrum of SNR levels are required to determine which prosodic type is more robust, and less susceptible to background noise, thus ensuring increased generalizability. By investigating the relative salience of linguistic and emotional prosody, researchers can provide insights into improving communication strategies in various populations who have difficulties with prosody processing, enhancing educational experiences, and gaining a deeper understanding of human cognitive and emotional processes.

## Figures and Tables

**Table 1:** Mean values (SD) of the acoustic measures for the prosodic stimuli: mean F0 (Hz), duration (msec), and mean intensity (dB).

Measure	Emotional Prosody	Lexical Tone
Mean F0 (Hz)	195.6 (76.1)	151.9 (46.3)
Duration (msec)	525.2 (185.6)	570.0 (84.6)
Mean intensity (dB)	77.7 (2.4)	78.0 (2.6)

## Data Availability

Publicly available datasets were analyzed in this study. This data can be found here: https://osf.io/r8nmk/?view_only=6ad5e69885ba48cea3ab69ee77ed84a8 (accessed on 13 April 2023).

## Supplementary Materials

The following supporting information can be downloaded at: https://www.mdpi.com/article/10.3390/bs13100800/s1, Supplemental Material S1: The full models with intercepts, coefficients, and error terms for accuracy and reaction time analyses; Table S1: Generalized linear mixed-effects model with task and condition as the fixed effects, and accuracy as the dependent variable (pairwise contrasts are indented); Table S2: Generalized linear mixed-effects model with task and condition as the fixed effects, and reaction time as the dependent variable (pairwise contrasts are indented).

## References

[1] Fairbanks G., Pronovost W. (1938). Vocal Pitch During Simulated Emotion. Science.

[2] Monrad-Krohn G.H. (1947). The Prosodic Quality of Speech and Its Disorders: A Brief Survey from a Neurologist’s Point of View. Acta Psychiatr. Scand..

[3] Cutler A., Pearson M., Johns-Lewis C. (2018). On the Analysis of Prosodic Turn-Taking Cues. Intonation in Discourse.

[4] Belyk M., Brown S. (2014). Perception of Affective and Linguistic Prosody: An ALE Meta-Analysis of Neuroimaging Studies. Soc. Cogn. Affect. Neurosci..

[5] Ding H., Zhang Y. (2023). Speech Prosody in Mental Disorders. Annu. Rev. Linguist..

[6] Fromkin V.A., Curtiss S., Hayes B.P., Hyams N., Keating P.A., Koopman H., Steriade D. (2000). Linguistics: An Introduction to Linguistic Theory.

[7] Liang B., Du Y. (2018). The Functional Neuroanatomy of Lexical Tone Perception: An Activation Likelihood Estimation Meta-Analysis. Front. Neurosci..

[8] Gandour J.T., Carlos G., Tomas R. (2009). Neural Substrates Underlying the Perception of Linguistic Prosody. Volume 2 Experimental Studies in Word and Sentence Prosody.

[9] Massaro D.W., Cohen M.M., Tseng C.-Y. (1985). The Evaluation and Integration of Pitch Height and Pitch Contour in Lexical Tone Perception in Mandarin Chinese. J. Chin. Linguist..

[10] Xu Y. (1997). Contextual Tonal Variations in Mandarin. J. Phon..

[11] Jongman A., Wang Y., Moore C.B., Sereno J.A., Bates E., Tan L.H., Tzeng O.J.L., Li P. (2006). Perception and Production of Mandarin Chinese Tones. The Handbook of East Asian Psycholinguistics: Volume 1: Chinese.

[12] Moore C.B., Jongman A. (1997). Speaker Normalization in the Perception of Mandarin Chinese Tones. J. Acoust. Soc. Am..

[13] Yu K., Zhou Y., Li L., Su J.A., Wang R., Li P. (2017). The Interaction between Phonological Information and Pitch Type at Pre-Attentive Stage: An ERP Study of Lexical Tones. Lang. Cogn. Neurosci..

[14] Tseng C.-Y., Massaro D.W., Cohen M.M., Kao H.S.R., Hoosain R. (1986). Lexical Tone Perception in Mandarin Chinese: Evaluation and Integration of Acoustic Features. Linguistics, Psychology, and the Chinese Language.

[15] Whalen D.H., Xu Y. (1992). Information for Mandarin Tones in the Amplitude Contour and in Brief Segments. Phonetica.

[16] Lin M.-C. (1988). The Acoustic Characteristics and Perceptual Cues of Tones in Standard Chinese [Putonghua Shengdiao De Shengxue Texing He Zhijue Zhengzhao]. Chin. Linguist. [Zhongguo Yuwen].

[17] Xu L., Tsai Y., Pfingst B.E. (2002). Features of Stimulation Affecting Tonal-Speech Perception: Implications for Cochlear Prostheses. J. Acoust. Soc. Am..

[18] Kong Y.-Y., Zeng F.-G. (2006). Temporal and Spectral Cues in Mandarin Tone Recognition. J. Acoust. Soc. Am..

[19] Krenmayr A., Qi B., Liu B., Liu H., Chen X., Han D., Schatzer R., Zierhofer C.M. (2011). Development of a Mandarin Tone Identification Test: Sensitivity Index *D′* as a Performance Measure for Individual Tones. Int. J. Audiol..

[20] Lee C.-Y., Tao L., Bond Z.S. (2013). Effects of Speaker Variability and Noise on Mandarin Tone Identification by Native and Non-Native Listeners. Speech Lang. Hear..

[21] Qi B., Mao Y., Liu J., Liu B., Xu L. (2017). Relative Contributions of Acoustic Temporal Fine Structure and Envelope Cues for Lexical Tone Perception in Noise. J. Acoust. Soc. Am..

[22] Wang X., Xu L. (2020). Mandarin Tone Perception in Multiple-Talker Babbles and Speech-Shaped Noise. J. Acoust. Soc. Am..

[23] Apoux F., Yoho S.E., Youngdahl C.L., Healy E.W. (2013). Role and Relative Contribution of Temporal Envelope and Fine Structure Cues in Sentence Recognition by Normal-Hearing Listeners. J. Acoust. Soc. Am..

[24] Morgan S.D. (2021). Comparing Emotion Recognition and Word Recognition in Background Noise. J. Speech Lang. Hear. Res..

[25] Lakshminarayanan K., Ben Shalom D., van Wassenhove V., Orbelo D., Houde J., Poeppel D. (2003). The Effect of Spectral Manipulations on the Identification of Affective and Linguistic Prosody. Brain Lang..

[26] van Zyl M., Hanekom J.J. (2011). Speech Perception in Noise: A Comparison between Sentence and Prosody Recognition. J. Hear. Sci..

[27] Krishnan A., Xu Y., Gandour J., Cariani P. (2005). Encoding of Pitch in the Human Brainstem Is Sensitive to Language Experience. Cogn. Brain Res..

[28] Klein D., Zatorre R.J., Milner B., Zhao V. (2001). A Cross-Linguistic PET Study of Tone Perception in Mandarin Chinese and English Speakers. NeuroImage.

[29] Braun B., Johnson E.K. (2011). Question or Tone 2? How Language Experience and Linguistic Function Guide Pitch Processing. J. Phon..

[30] Ekman P. (1992). An Argument for Basic Emotions. Cogn. Emot..

[31] Sauter D.A., Eisner F., Ekman P., Scott S.K. (2010). Cross-Cultural Recognition of Basic Emotions through Nonverbal Emotional Vocalizations. Proc. Natl. Acad. Sci. USA.

[32] Scherer K.R., Banse R., Wallbott H.G. (2001). Emotion Inferences from Vocal Expression Correlate across Languages and Cultures. J. Cross Cult. Psychol..

[33] Pell M.D., Monetta L., Paulmann S., Kotz S.A. (2009). Recognizing Emotions in a Foreign Language. J. Nonverbal Behav..

[34] Scherer K.R. (1986). Vocal Affect Expression: A Review and a Model for Future Research. Psychol. Bull..

[35] Liu P., Pell M.D. Processing Emotional Prosody in Mandarin Chinese: A Cross-Language Comparison. Proceedings of the International Conference on Speech Prosody 2014.

[36] Bryant G.A., Barrett H.C. (2008). Vocal Emotion Recognition across Disparate Cultures. J. Cogn. Cult..

[37] Wang T., Lee Y.-c., Ma Q. (2018). Within and across-Language Comparison of Vocal Emotions in Mandarin and English. Appl. Sci..

[38] Banse R., Scherer K.R. (1996). Acoustic Profiles in Vocal Emotion Expression. J. Pers. Soc. Psychol..

[39] Dupuis K., Pichora-Fuller M.K. (2014). Intelligibility of Emotional Speech in Younger and Older Adults. Ear Hear..

[40] Castro S.L., Lima C.F. (2010). Recognizing Emotions in Spoken Language: A Validated Set of Portuguese Sentences and Pseudosentences for Research on Emotional Prosody. Behav. Res. Methods.

[41] Murray I.R., Arnott J.L. (1993). Toward the Simulation of Emotion in Synthetic Speech: A Review of the Literature on Human Vocal Emotion. J. Acoust. Soc. Am..

[42] Juslin P.N., Laukka P. (2003). Communication of Emotions in Vocal Expression and Music Performance: Different Channels, Same Code?. Psychol. Bull..

[43] Zhang S., Sun F., Zhang J., Tan Y., Cao J., Yu W. (2008). Emotion Recognition in Chinese Natural Speech by Combining Prosody and Voice Quality Features.

[44] Hirst D., Wakefield J., Li H.Y. Does Lexical Tone Restrict the Paralinguistic Use of Pitch? Comparing Melody Metrics for English, French, Mandarin and Cantonese. Proceedings of the International Conference on the Phonetics of Languages in China.

[45] Zhao X., Zhang S., Lei B. (2014). Robust Emotion Recognition in Noisy Speech Via Sparse Representation. Neural Comput. Appl..

[46] Schuller B., Arsic D., Wallhoff F., Rigoll G. Emotion Recognition in the Noise Applying Large Acoustic Feature Sets. Proceedings of the Speech Prosody 2006.

[47] Scharenborg O., Kakouros S., Koemans J. The Effect of Noise on Emotion Perception in an Unknown Language. Proceedings of the International Conference on Speech Prosody 2018.

[48] Parada-Cabaleiro E., Baird A., Batliner A., Cummins N., Hantke S., Schuller B. The Perception of Emotions in Noisified Non-Sense Speech. Proceedings of the Interspeech 2017.

[49] Zhang M., Ding H. Impact of Background Noise and Contribution of Visual Information in Emotion Identification by Native Mandarin Speakers. Proceedings of the Interspeech 2022.

[50] Parada-Cabaleiro E., Batliner A., Baird A., Schuller B. (2020). The Perception of Emotional Cues by Children in Artificial Background Noise. Int. J. Speech Technol..

[51] Luo X. (2016). Talker Variability Effects on Vocal Emotion Recognition in Acoustic and Simulated Electric Hearing. J. Acoust. Soc. Am..

[52] Hockett C. (1967). The Quantification of Functional Load. Word.

[53] Ross E.D., Edmondson J.A., Seibert G.B. (1986). The Effect of Affect on Various Acoustic Measures of Prosody in Tone and Non-Tone Languages: A Comparison Based on Computer Analysis of Voice. J. Phon..

[54] Xu Y., Katz W.F., Assmann P.F. (2019). Prosody, Tone and Intonation. The Routledge Handbook of Phonetics.

[55] Scherer K.R., Wallbott H.G. (1994). Evidence for Universality and Cultural Variation of Differential Emotion Response Patterning. J. Pers. Soc. Psychol..

[56] Viswanathan V., Shinn-Cunningham B.G., Heinz M.G. (2021). Temporal Fine Structure Influences Voicing Confusions for Consonant Identification in Multi-Talker Babble. J. Acoust. Soc. Am..

[57] Cherry E.C. (1953). Some Experiments on the Recognition of Speech, with One and with Two Ears. J. Acoust. Soc. Am..

[58] Statista Search Department The Most Spoken Languages Worldwide 2023, by Speakers in Millions. 16 June 2023. Distributed by Statista. https://www.statista.com/statistics/266808/the-most-spoken-languages-worldwide/.

[59] Koerner T.K., Zhang Y. (2018). Differential Effects of Hearing Impairment and Age on Electrophysiological and Behavioral Measures of Speech in Noise. Hear. Res..

[60] Ameka F. (1992). Interjections: The Universal yet Neglected Part of Speech. J. Pragmat..

[61] Howie J.M. (1974). On the Domain of Tone in Mandarin. Phonetica.

[62] Xu Y. (2013). Prosodypro—A Tool for Large-Scale Systematic Prosody Analysis. Tools and Resources for the Analysis of Speech Prosody.

[63] Boersma P., Weenink D. (2018). Praat: Doing Phonetics by Computer, 6.0.37 [Computer Program]. http://www.fon.hum.uva.nl/praat.

[64] Wang Y., Jongman A., Sereno J.A. (2003). Acoustic and Perceptual Evaluation of Mandarin Tone Productions before and after Perceptual Training. J. Acoust. Soc. Am..

[65] Liu S., Samuel A.G. (2004). Perception of Mandarin Lexical Tones When F0 Information Is Neutralized. Lang. Speech.

[66] Li A. (2015). Emotional Intonation and Its Boundary Tones in Chinese. Encoding and Decoding of Emotional Speech: A Cross-Cultural and Multimodal Study between Chinese and Japanese.

[67] Chen F., Hu Y., Yuan M. (2015). Evaluation of Noise Reduction Methods for Sentence Recognition by Mandarin-Speaking Cochlear Implant Listeners. Ear Hear..

[68] Bates D., Mächler M., Bolker B., Walker S. (2015). Fitting Linear Mixed-Effects Models Using Lme4. J. Stat. Softw..

[69] Lo S., Andrews S. (2015). To Transform or Not to Transform: Using Generalized Linear Mixed Models to Analyse Reaction Time Data. Front. Psychol..

[70] Baayen R.H., Milin P. (2010). Analyzing Reaction Times. Int. J. Psychol. Res..

[71] Chien Y.F., Sereno J.A., Zhang J. (2017). What’s in a Word: Observing the Contribution of Underlying and Surface Representations. Lang. Speech.

[72] Lenth R. (2020). Emmeans: Estimated Marginal Means, Aka Leastsquares Means.

[73] Brungart D.S., Simpson B.D., Ericson M.A., Scott K.R. (2001). Informational and Energetic Masking Effects in the Perception of Multiple Simultaneous Talkers. J. Acoust. Soc. Am..

[74] Scott S.K., McGettigan C. (2013). The Neural Processing of Masked Speech. Hear. Res..

[75] Shinn-Cunningham B.G. (2008). Object-Based Auditory and Visual Attention. Trends Cogn. Sci..

[76] Mattys S.L., Brooks J., Cooke M. (2009). Recognizing Speech under a Processing Load: Dissociating Energetic from Informational Factors. Cogn. Psychol..

[77] Rosen S., Souza P., Ekelund C., Majeed A. (2013). Listening to Speech in a Background of Other Talkers: Effects of Talker Number and Noise Vocoding. J. Acoust. Soc. Am..

[78] Wang T., Ding H., Kuang J., Ma Q. Mapping Emotions into Acoustic Space: The Role of Voice Quality. Proceedings of the Interspeech 2014.

[79] Ingrisano D.R.-S., Perry C.K., Jepson K.R. (1998). Environmental Noise. Am. J. Speech Lang. Pathol..

[80] Perry C.K., Ingrisano D.R.S., Palmer M.A., McDonald E.J. (2000). Effects of Environmental Noise on Computer-Derived Voice Estimates from Female Speakers. J. Voice.

[81] Wang T., Lee Y.-C. (2015). Does Restriction of Pitch Variation Affect the Perception of Vocal Emotions in Mandarin Chinese?. J. Acoust. Soc. Am..

[82] Schirmer A., Kotz S.A. (2006). Beyond the Right Hemisphere: Brain Mechanisms Mediating Vocal Emotional Processing. Trends Cogn. Sci..

[83] Fugate J.M.B. (2013). Categorical Perception for Emotional Faces. Emot. Rev..

[84] Singh L., Fu C.S.L. (2016). A New View of Language Development: The Acquisition of Lexical Tone. Child Dev..

[85] Yeung H.H., Chen K.H., Werker J.F. (2013). When Does Native Language Input Affect Phonetic Perception? The Precocious Case of Lexical Tone. J. Mem. Lang..

[86] Shablack H., Lindquist K.A., LoBue V., Pérez-Edgar K., Buss K.A. (2019). The Role of Language in Emotional Development. Handbook of Emotional Development.

[87] Morningstar M., Venticinque J., Nelson E.E. (2019). Differences in Adult and Adolescent Listeners’ Ratings of Valence and Arousal in Emotional Prosody. Cogn. Emot..

[88] Zhao L., Sloggett S., Chodroff E. Top-Down and Bottom-up Processing of Familiar and Unfamiliar Mandarin Dialect Tone Systems. Proceedings of the Speech Prosody 2022.

[89] Zhao T.C., Kuhl P.K. (2015). Top-Down Linguistic Categories Dominate over Bottom-up Acoustics in Lexical Tone Processing. J. Acoust. Soc. Am..

[90] Malins J.G., Gao D., Tao R., Booth J.R., Shu H., Joanisse M.F., Liu L., Desroches A.S. (2014). Developmental Differences in the Influence of Phonological Similarity on Spoken Word Processing in Mandarin Chinese. Brain Lang..

[91] Shuai L., Gong T. (2014). Temporal Relation between Top-Down and Bottom-up Processing in Lexical Tone Perception. Front. Behav. Neurosci..

[92] Başkent D. (2012). Effect of Speech Degradation on Top-Down Repair: Phonemic Restoration with Simulations of Cochlear Implants and Combined Electric–Acoustic Stimulation. J. Assoc. Res. Otolaryngol..

[93] Wang J., Shu H., Zhang L., Liu Z., Zhang Y. (2013). The Roles of Fundamental Frequency Contours and Sentence Context in Mandarin Chinese Speech Intelligibility. J. Acoust. Soc. Am..

[94] Sammler D., Grosbras M.-H., Anwander A., Bestelmeyer P.E.G., Belin P. (2015). Dorsal and Ventral Pathways for Prosody. Curr. Biol..

[95] Hochmuth S., Kollmeier B., Brand T., Jürgens T. (2015). Influence of Noise Type on Speech Reception Thresholds across Four Languages Measured with Matrix Sentence Tests. Int. J. Audiol..

[96] Kozou H., Kujala T., Shtyrov Y., Toppila E., Starck J., Alku P., Näätänen R. (2005). The Effect of Different Noise Types on the Speech and Non-Speech Elicited Mismatch Negativity. Hear. Res..

[97] Coulson S. (2023). Sensorimotor Account of Multimodal Prosody. PsyArXiv.

[98] Holler J., Levinson S.C. (2019). Multimodal Language Processing in Human Communication. Trends Cogn. Sci..

[99] Bryant G.A. (2022). Vocal Communication across Cultures: Theoretical and Methodological Issues. Philos. Trans. R. Soc. Lond. B Biol. Sci..

[100] Lecumberri M.L.G., Cooke M., Cutler A. (2010). Non-Native Speech Perception in Adverse Conditions: A Review. Speech Commun..

[101] Liu P., Rigoulot S., Pell M.D. (2015). Cultural Differences in on-Line Sensitivity to Emotional Voices: Comparing East and West. Front. Hum. Neurosci..

[102] Gordon-Salant S., Fitzgibbons P.J. (2004). Effects of Stimulus and Noise Rate Variability on Speech Perception by Younger and Older Adults. J. Acoust. Soc. Am..

[103] Goossens T., Vercammen C., Wouters J., van Wieringen A. (2017). Masked Speech Perception across the Adult Lifespan: Impact of Age and Hearing Impairment. Hear. Res..

[104] Van Engen K.J., Phelps J.E., Smiljanic R., Chandrasekaran B. (2014). Enhancing Speech Intelligibility: Interactions among Context, Modality, Speech Style, and Masker. J. Speech Lang. Hear. Res..

[105] Scott S.K., Rosen S., Wickham L., Wise R.J.S. (2004). A Positron Emission Tomography Study of the Neural Basis of Informational and Energetic Masking Effects in Speech Perception. J. Acoust. Soc. Am..

[106] Nygaard L.C., Queen J.S. (2008). Communicating Emotion: Linking Affective Prosody and Word Meaning. J. Exp. Psychol. Hum. Percept. Perform..

[107] Wilson D., Wharton T. (2006). Relevance and Prosody. J. Pragmat..

[108] Frühholz S., Trost W., Kotz S.A. (2016). The Sound of Emotions—Towards a Unifying Neural Network Perspective of Affective Sound Processing. Neurosci. Biobehav. Rev..

[109] Grandjean D. (2021). Brain Networks of Emotional Prosody Processing. Emot. Rev..

[110] Jiang A., Yang J., Yang Y. (2014). Mmn Responses During Implicit Processing of Changes in Emotional Prosody: An ERP Study Using Chinese Pseudo-Syllables. Cogn. Neurodyn..

[111] Lin Y., Fan X., Chen Y., Zhang H., Chen F., Zhang H., Ding H., Zhang Y. (2022). Neurocognitive Dynamics of Prosodic Salience over Semantics During Explicit and Implicit Processing of Basic Emotions in Spoken Words. Brain Sci..

[112] Mauchand M., Caballero J.A., Jiang X., Pell M.D. (2021). Immediate Online Use of Prosody Reveals the Ironic Intentions of a Speaker: Neurophysiological Evidence. Cogn. Affect. Behav. Neurosci..

[113] Chen Y., Tang E., Ding H., Zhang Y. (2022). Auditory Pitch Perception in Autism Spectrum Disorder: A Systematic Review and Meta-Analysis. J. Speech Lang. Hear. Res..

[114] Zhang L., Xia Z., Zhao Y., Shu H., Zhang Y. (2023). Recent Advances in Chinese Developmental Dyslexia. Annu. Rev. Linguist..

[115] Zhang M., Xu S., Chen Y., Lin Y., Ding H., Zhang Y. (2022). Recognition of Affective Prosody in Autism Spectrum Conditions: A Systematic Review and Meta-Analysis. Autism.

[116] Seddoh S.A. (2002). How Discrete or Independent Are “Affective Prosody”and “Linguistic Prosody”?. Aphasiology.

[117] Ben-David B.M., Gal-Rosenblum S., van Lieshout P.H., Shakuf V. (2019). Age-Related Differences in the Perception of Emotion in Spoken Language: The Relative Roles of Prosody and Semantics. J. Speech Lang. Hear. Res..

