# Supplementary material for: Evaluating the Relative Perceptual Salience of Linguistic and Emotional Prosody in Quiet and Noisy Contexts
Source: Behav Sci (Basel). 2023 Sep 26;13(10):800. doi: 10.3390/bs13100800 (PMC10603920; doi:10.3390/bs13100800)
Supplement: Supplementary file 1 [file behavsci-13-00800-s001.zip › behavsci-2625118_Revised Supplemental Table S1.pdf]

Supplemental Table S1. Generalized linear mixed-effects model with task and condition as the fixed effects, and accuracy as the dependent variable (pairwise contrasts are indented).

Parameter	Estimate	Standard error (*SE*)	*z* ratio	*p*	Cohen's *d* [95% CI]
**Task** ($\chi 2(2) = 199.46$, $p < .001$, Cohen's $w = 2.23$)					
emotion vs. tone	-0.90	0.07	-12.92	< .0001	-1.06 [-1.22, -0.90]
**Condition** ($\chi 2(2) = 1752.9$, $p < .001$, $w = 6.62$)					
quiet vs. noise	2.38	0.07	33.90	< .0001	2.81 [2.65, 2.97]
**Task * Condition** ($\chi 2(1) = 27.75$, $p < .001$, $w = 0.83$)					
emotion (quiet vs. noise)	2.03	0.08	26.45	< .0001	2.39 [2.21, 2.57]
tone (quiet vs. noise)	2.74	0.12	23.42	< .0001	3.23 [2.96, 3.50]
quiet (emotion vs. tone)	-1.26	0.13	-9.97	< .0001	-1.48 [-1.77, -1.19]
noise (emotion vs. tone)	-0.54	0.06	-9.16	< .0001	-0.64 [-0.78, -0.50]

Note. The emotion recognition task and the quiet condition were used as the default level of task and condition respectively.
