# Supplementary material for: Evaluating the Relative Perceptual Salience of Linguistic and Emotional Prosody in Quiet and Noisy Contexts
Source: Behav Sci (Basel). 2023 Sep 26;13(10):800. doi: 10.3390/bs13100800 (PMC10603920; doi:10.3390/bs13100800)
Supplement: Supplementary file 1 [file behavsci-13-00800-s001.zip › behavsci-2625118_Revised Supplemental Table S2.pdf]

Supplemental Table S2. Generalized linear mixed-effects model with task and condition as the fixed effects, and reaction time as the dependent variable (pairwise contrasts are indented).

Parameter	Estimate	Standard error (*SE*)	*z* ratio	*p*	Cohen's *d* [95% CI]
**Task** ($\chi 2(2) = 20.16$, $p < .001$, Cohen's $w = .71$)					
emotion vs. tone	0.04	0.009	4.27	< .0001	0.05 [0.03, 0.08]
**Condition** ($\chi 2(2) = 408.73$, $p < .001$, $w = 3.20$)					
quiet vs. noise	-0.18	0.009	-20.35	< .0001	-0.24 [-0.27, -0.22]
**Task * Condition** ($\chi 2(1) = 5.17$, $p = .02$, $w = .36$)					
emotion (quiet vs. noise)	-0.20	0.013	-15.44	< .0001	-0.27 [-0.31, -0.24]
tone (quiet vs. noise)	-0.16	0.012	-13.35	< .0001	-0.22 [-0.25, -0.18]
quiet (emotion vs. tone)	0.02	0.011	1.61	0.374	0.02 [-0.01, 0.05]
noise (emotion vs. tone)	0.06	0.014	4.20	0.0002	0.08 [0.04, 0.11]

Note. The emotion recognition task and the quiet condition were used as the default level of task and condition respectively.
