# Supplementary material for: Evaluating the Relative Perceptual Salience of Linguistic and Emotional Prosody in Quiet and Noisy Contexts
Source: Behav Sci (Basel). 2023 Sep 26;13(10):800. doi: 10.3390/bs13100800 (PMC10603920; doi:10.3390/bs13100800)
Supplement: Supplementary file 1 [file behavsci-13-00800-s001.zip › behavsci-2625118_Supplemental Material S1.pdf]

Supplemental Material S1. The full models with intercepts, coefficients, and error terms for accuracy and reaction time analyses.

$$Accuracy_{ij} = \beta_0 + (\beta_1 \times task) + (\beta_2 \times condition) + (\beta_3 \times task * condition) + b_{0i} + b_{1j} + \varepsilon_{ij} \quad (1)$$

$$Reaction\ time_{ij} = \beta_0 + (\beta_1 \times task) + (\beta_2 \times condition) + (\beta_3 \times task * condition) + b_{0i} + b_{1j} + \varepsilon_{ij} \quad (2)$$

In these models, β_0 represented the intercept, which was the predicted outcome when all other predictors were equal to 0. β_1, β_2, and β_3 represented the coefficients for the two fixed factors and their interaction respectively. These coefficients reflected how much the outcome variable changed relative to a unit of change in the corresponding predictors. The random intercepts were represented as b_{0i} and b_{1j}, where i varied according to decoder participants and j varied according to test items. An error term (ε) was also included to account for the distance between the predicted value and the actual data point (i.e., residual).
